# Tumor Budding as a Marker for Poor Prognosis and Epithelial–Mesenchymal Transition in Lung Cancer: A Systematic Review and Meta-Analysis

**DOI:** 10.3389/fonc.2022.828999

**Published:** 2022-06-02

**Authors:** Nishant Thakur, Muhammad Joan Ailia, Yosep Chong, Ok Ran Shin, Kwangil Yim

**Affiliations:** Department of Hospital Pathology, The Catholic University of Korea, Seoul, South Korea

**Keywords:** tumor budding, meta-analysis, lung cancer, prognosis, epithelial–mesenchymal transition

## Abstract

**Introduction:**

Currently, tumor budding (TB) is considered to predict the prognosis of patients. The prognostic significance of TB has also been explored in patients with lung cancer, but has not been fully clarified. In the present meta-analysis, we evaluated the prognostic significance, clinicopathological value, and relationship with epithelial–mesenchymal transition (EMT) of TB in lung cancer.

**Methods:**

The MEDLINE, EMBASE, and Cochrane databases were searched up to July 7, 2021, for the relevant articles that showed the relationship between TB and prognosis in patients with lung cancer. For statistical analysis, we used pooled hazard ratios (HRs) with their corresponding 95% confidence intervals (CIs) to assess the correlation between high-grade TB expression and overall survival (OS), disease-free survival (DFS), progression-free survival (PFS), clinicopathological factors, and EMT markers.

**Results:**

A total of 3,784 patients from 10 independent studies were included in the statistical analysis. Our results indicated that high-grade TB was significantly associated with poor OS [HR 1.64 (95% CI, 1.43–1.87)] and DFS [HR 1.65 (95% CI, 1.22–2.25)]. In terms of clinicopathological characteristics, high-grade TB was associated with larger tumor size, higher T and N stage, pleural invasion, vascular invasion, lymphatic invasion, and severe nuclear atypia. Interestingly, smoking showed significant association with high-grade TB, despite the fact that previous studies could not show a significant relationship between them. Furthermore, through our systematic analysis, high-grade TB showed a significant relationship with EMT markers.

**Conclusion:**

Our findings indicate that high-grade TB is associated with a worse prognosis in patients with lung cancer. TB evaluation should be implemented in routine pathological diagnosis, which may guide the patient’s treatment.

## Introduction

Lung cancer is one of the most aggressive cancers and is the leading cause of cancer mortality worldwide (1, 2). The 5-year survival rate was 63% for patients with localized stage, while it was less than 5% for those with advanced metastatic stage (1, 3). The introduction of effective treatment strategies, including surgery, radiotherapy, targeted therapy, and immunotherapies, has recently improved the clinical outcomes of lung cancer patients (4). Despite advancements in the present treatment, most patients commonly experience recurrence and still have a poor prognosis (5). Adjuvant chemotherapy is essential for some patients with resected lung cancer to attain improved clinical outcomes; however, it is unclear which patients benefit from adjuvant chemotherapy (6). Therefore, it is important to predict an accurate prognosis.

Recently, tumor budding (TB) has received the attention of pathologists and is considered to predict the prognosis of patients (7). TB is generated by a process that involves detached isolated malignant cells or clusters of up to four cancer cells that move to the stromal region by dissociating at the invasive front. TB is part of the tumor microenvironment (TME) and is related to epithelial–mesenchymal transition (EMT) (7, 8). The prognostic significance of TB has been explored in solid cancers (7), such as colon cancer (9, 10), gastric cancer (11, 12), gynecologic cancer (13, 14), and pancreatic cancer (15). Moreover, the prognostic significance of TBs and an association with EMT have also been explored in patients with lung cancer (16–25), but it has not been fully clarified. In addition, various methods have been used to analyze TBs in lung cancer tissues. However, standardization of the TB assessment method is needed (16–25).

Thus, the objective of the present study was to perform a meta-analysis and systematically evaluate the prognostic significance, clinicopathological impact of TBs, assessment methods, and the relationship between EMT and TBs in patients with lung cancer.

## Materials and Methods

We conducted this meta-analysis according to the following guidelines set out by the PRISMA (Preferred Reporting Items for Systematic Reviews and Meta-analysis) statement (26) and also submitted the protocol at the PROSPERO database (CRD42021271951).

### Search Strategy

The present study was approved by the Institutional Review Board of the Catholic University of Korea, College of Medicine (UC21ZISI0060). MEDLINE, EMBASE, and Cochrane Library were used for relevant articles written in English that were published up to July 7, 2021. The search terminologies were summarized in **Supplementary Table 1**. Next, references were manually searched by cross-referencing key articles. EndNote X20 (Bld 10136, Thomson Reuters, New York, NY, USA) was used to retrieve and manage the records.

### Inclusion and Exclusion Criteria

In this meta-analysis, eligible studies were required to meet the following inclusion criteria: (1) the relationship between TB and survival rates of patients was evaluated; (2) TB was diagnosed accurately by histopathology with precise microscopic demonstration; (3) studies provided enough information to estimate survival, clinicopathological parameters, or EMT markers; and (4) articles were written in English. In case of a lack of hazard ratio (HR), we used the Kaplan–Meier curve data to calculate the HR using the method of Parmar et al. (27). Exclusion criteria were as follows: (1) duplicated studies, reviews, case reports, and letters; and (2) studies that did not show an association between TB and survival, clinicopathological factors, or EMT markers.

### Data Extraction and Assessment of Study Quality

NT and KY extracted the data; if any disagreement occurred during the process, it was resolved by consensus or senior pathologists (YC and OS). The detailed clinicopathological parameters information was extracted from all studies and described in **Table 1** and **Supplementary Table 3**. The Newcastle–Ottawa Scale system was exploited to evaluate the quality analysis of all studies (29).

**Table 1:** Main characteristics of all lung cancer included studies.

Histological subtype	Author/year/reference	Ethnicity	Patient number	Staining method	Assessment method/cutoff	Field of view	HR (95% CI)	NOS score
LSCC	Taira, 2011 (20)	Asian	237	H&E Pan-cytokeratin (AE1/AE3)^*^	TB-YN >0	×200	OS: 1.597 (1.069–2.384)	8
	Masuda, 2012 (18)	Asian	103	H&E	TB-YN >0	×200	OS: 2.766 (1.497–5.109)	8
	Kadota (1), 2014 (16)	Caucasian	485	H&E	TB-1HPF ≥10	×200	OS: 1.33 (1.03–1.70)	8
	Weichert, 2015 (22)	Caucasian	440	H&E	TB-YN >0 TB-1HPF ≥5 TB-10HPF ≥15	×400 (0.24 mm^2^)	OS:2.40 (1.42–4.04) DFS: 1.60 (1.04–2.46)	8
	Zhao, 2015 (24)	Asian	132	H&E	TB-YN >0	×400	OS: 0.466 (0.272–0.799)	7
	Kadota (3), 2017 (25)	Asian	216	H&E	TB-1HPF ≥10	×200	DFS: 1.15 (1.10–1.21)	7
	Neppl, 2020 (19)	Caucasian	354	H&E and Pan-cytokeratin (AE1/AE3)^**^	TB-1HPF ≥5	×200 (0.785 mm^2^)	OS: 1.581(1.186–2.108) DFS: 1.710(1.11–2.632) PFS: 1.457(1.123–1.89)	7
LADC	Yamaguchi, 2010 (23)	Asian	665	H&E Pan-cytokeratin (AE1/AE3)^*^	TB-1HPF ≥5	×200	OS: 1.872 (1.062–3.298)	8
	Kadota (2), 2015 (17)	Caucasian	1,038	H&E	TB-1HPF ≥5	×200	OS: 1.61 (1.13–2.29)	8
	Ammour, 2017 (28)	Caucasian	12 (3)^†^	Pan-cytokeratin (AE1/AE3)^†^	–	×200	–	2
	Vasilijević, 2021 (21)	Caucasian	114	H&E	TB-YN >0	×200	OS: 1.47 (0.80–2.71)	7

EMT, epithelial–mesenchymal transition; HR, hazard ratio; NOS, Newcastle–Ottawa score system; LADC, lung adenocarcinoma; LSCC, lung squamous cell carcinoma; Mag., magnification; TB, tumor budding; AC, adjuvant chemotherapy; OS, overall survival; DFS, disease-free survival; PFS, progression-free survival; RFS, recurrence-free survival; H&E, hematoxylin and eosin; IHC, immunohistochemistry. ^*^Pan-cytokeratin (AE1/AE3) is used to assist in assessing tumor budding. ^**^Authors compare the assessment methods for tumor budding between H&E and pan-cytokeratin (AE1/AE3). ^†^Ammour et al. collect pancreatic cancers, breast cancers, colorectal cancers, and lung cancers (3 cases in each cancer); three-dimensional reconstruction of slides was performed for the evaluation of epithelial–mesenchymal transition and histomorphological characteristics.

### Statistical Analysis

Statistical analysis was conducted using the Review Manager software, version 5.3 (30). The detailed description of the statistical analysis is explained in our previous article (31).

## Results

### Eligible Studies

Only 11 articles were included in the full-text review (**Supplementary Figure 1**) (16–25, 28), which included prognosis-based studies (*n* = 10) (16–25) and EMT marker-based studies (*n* = 4) (20, 23, 25, 28). Eventually, only 10 articles fulfilled the inclusion criteria for this meta-analysis (**Supplementary Figure 1**) (16–25).

### Study Characteristics

The main characteristics of all ten studies included in the meta-analysis based on TB are shown in **Table 1**) (16–25). Regarding histological subtype, seven articles focused on lung squamous cell carcinoma (LSCC) while three focused on lung adenocarcinoma (LADC) (**Table 1**). The total number of patients was 3,784 with stages I–IV. Hematoxylin and eosin (H&E) and pan-cytokeratin staining were used as detection methods. Moreover, four articles studied EMT markers (20, 23, 25, 28) while one article studied immune cell markers in relation to TB (17). Nine articles showed a correlation between TB and overall survival (OS), three were related to disease-free survival (DFS), and one was related to progression-free survival (PFS). Most of the studies achieved NOS scores higher than seven (**Table 1**; **Supplementary Tables 2, 3**).

### Relationship Between TB Expression and OS and DFS in Patients With Lung Cancer

**Figure 1:**
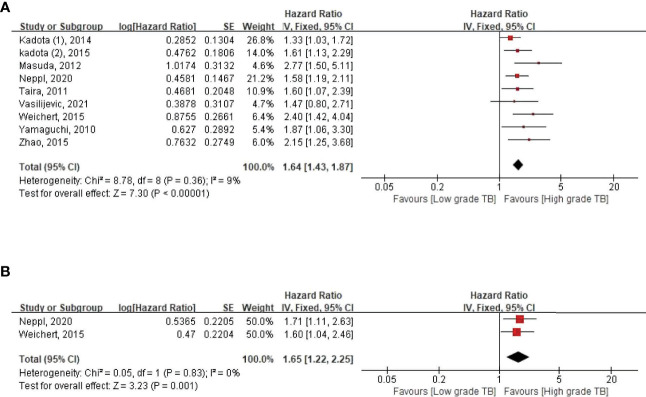
Pooled hazard ratios for **(A)** overall survival and **(B)** disease-free survival according to the tumor budding expression.

**Figure 2:**
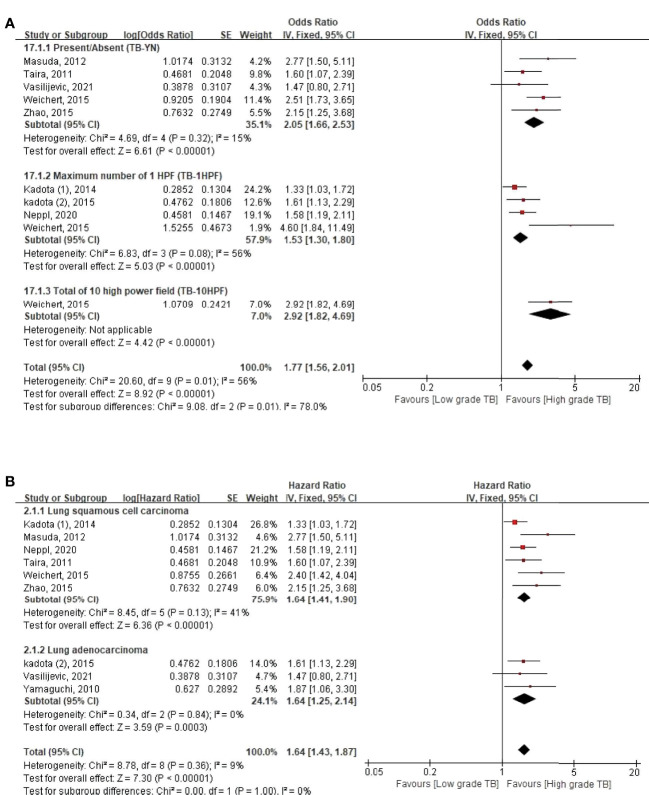
Subgroup hazard ratios analyzing the tumor budding expression for overall survival, by **(A)** assessment methods and **(B)** histologic type of lung cancer.

We evaluated the correlation between TB and OS among 3,568 patients with lung cancer from nine studies (**Table 1**) (16–25). During our pooled HR analysis, we found that the weight of Kadota et al. (3, 25) was too high (85.4%) compared to the relatively small sample size (*n* = 216, **Table 1**) with high heterogeneity (*I* ^2^ = 73%) (**Supplementary Figure 2A**) (25). Therefore, we decided to remove Kadota et al. (3) for the final analysis, and then the heterogeneity was reduced (*I^2^* = 9%) (**Figure 1A**). The pooled HR for OS demonstrated that high-grade TB was significantly associated with poor OS (HR 1.64, 95% CI 1.43–1.87; *p* < 0.00001) (**Figure 1A**). To examine the heterogeneity of these studies, subgroup analysis was performed based on four characteristics: assessment methods, histological subtype, ethnicity, and univariate versus multivariate analyses (**Figure 2** and **Supplementary Figure 3**). In every subgroup analysis, high TBs were associated with poor OS (**Figure 2A** and **Supplementary Figure 3**). In addition, heterogeneities were relatively low (*I* ^2^ < 50%), except in the univariate analysis subgroup (*I* ^2^ = 71%) (**Supplementary Figure 3**).

In DFS, three studies were included in the meta-analysis (19, 22, 25) and also found that the weight of Kadota et al. (3, 25) was too high (97.9%) and showed high heterogeneity (63%) (**Supplementary Figure 2B**). However, after removing this study, the heterogeneity was reduced (*I* ^2^ = 0%), and we found that high-grade TB was a poor DFS marker (HR 1.65, 95% CI 1.22–2.24, *p* = 0.001) (**Figure 1B**).

### Relationship Between TB Expression and Clinicopathological Parameters

TB-related clinicopathological parameters of all studies included in the meta-analysis are shown in **Supplementary Table 4**. The elevated expression of TB was significantly associated with clinicopathological parameters such as larger tumor size (≤30 vs. >30 mm), higher T stage (1–2 vs. 3–4), presence of lymph node metastasis, higher pathological stage (I–II vs. III–IV), presence of pleural invasion, presence of lymphatic invasion, presence of vascular invasion, nuclear atypia (mild-moderate vs. severe), and smoking (never vs. ever) (**Supplementary Table 5** and **Supplementary Figures 4–6**).

### TB Assessment Methods: Present/Absent (TB-YN), Maximum Number in One High-Power Field (TB-1HPF), and Total Number in 10 High-Power Fields (TB-10HPF)

**Figure 3:**
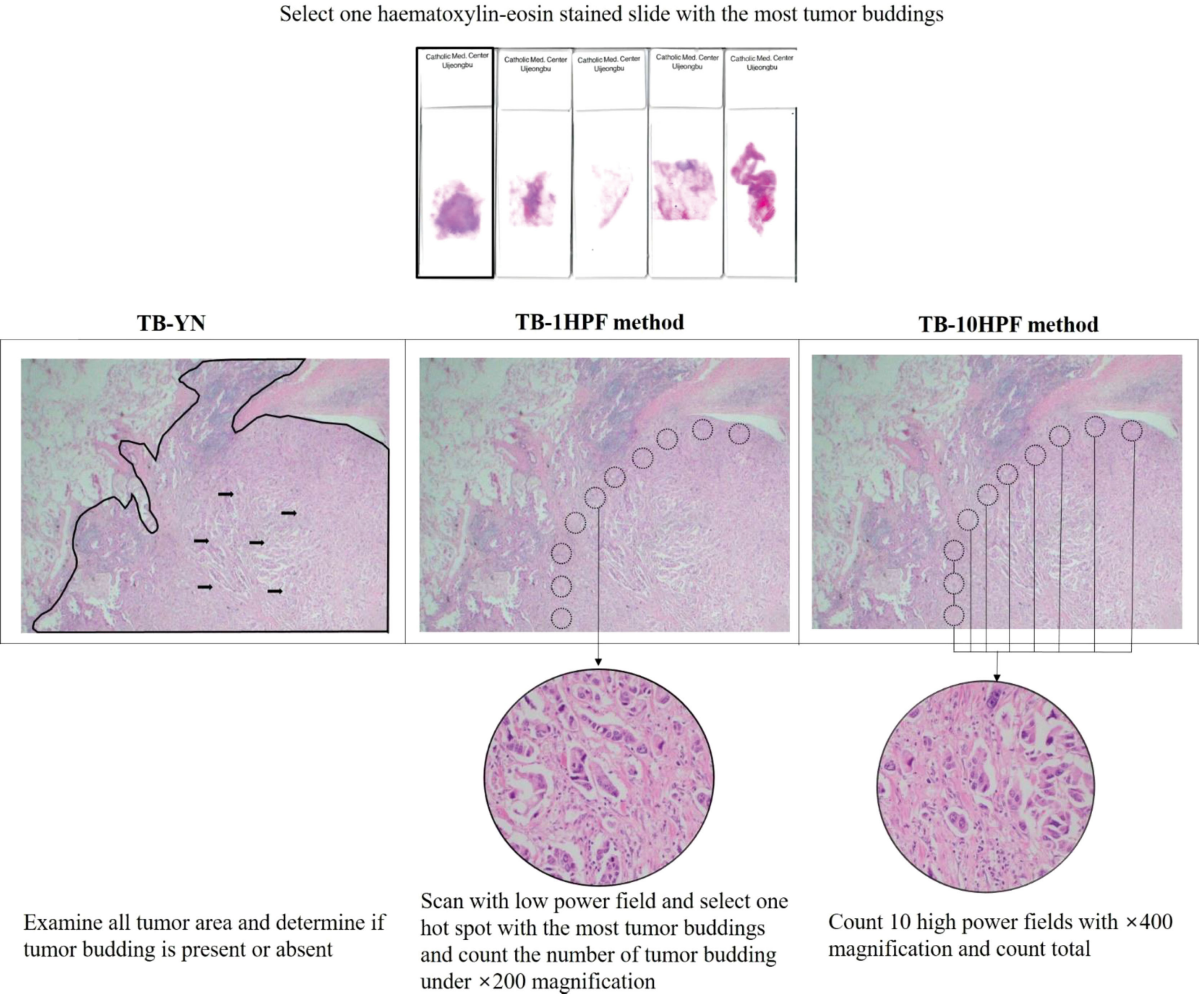
Description of three representative tumor budding assessment methods.

Three kinds of assessment methods were used to evaluate TB (**Figure 3**). First, in the TB-YN, the studies classified tumors into the presence or absence of TBs (18, 20–22, 24). Second, in the TB-1HPF, they searched with a low-power field and selected one “hotspot” in ×200 magnification and counted the number of TBs (16, 17, 22, 23, 25). In another study in the same group, Neppl et al. used the International TB Consensus Conference (ITBCC) method. According to this method, after selecting the hotspot area by searching with a low-power field, the number of TBs was counted at ×200 magnification. It was converted to fit the count from a 0.785 mm^2^ field area (19). Third, in TB-10HPF, the total number of TBs in 10 high-power fields was scanned at ×400 magnification (22). The Weichert et al. study used all three methods together (22). Therefore, we used all of these methods in our statistical analysis (**Table 1** and **Figure 3**).

### Relationship Between TB Expression With EMT and Immune Cell Infiltration

To determine the association of EMT with TB, we found four studies on EMT (20, 23, 25, 28). The detailed findings of each study are summarized in **Table 2**. In all EMT studies, we found decreased expression of E-cadherin, β-catenin, and geminin and increased expression of vimentin, laminin 5γ2, and ZEB1 in TB (**Table 2**). Also, in KRAS wild-type lung cancers, TB was significantly increased (**Table 2**). Interestingly, Ammour et al. found that TB can be divided into connected TB or isolated TB into the main mass, but they could not find any difference in the EMT feature. Instead, the total cell number per tumor cluster was the crucial element that change the morphology and EMT marker expression with the series of an event. The loss of membranous E-cadherin in the cutoff of 9 cells per cluster, the shift of membranous to cytoplasmic E-cadherin staining found at 8 cells, and then an increase in nuclear ZEB1 at 7 cells were observed. Subsequently, morphological changes were also seen with different cutoff values of cells per cluster (28). Overall, all these studies demonstrated that EMT was significantly associated with TB, which indicates that TB was a morphologic marker for EMT in lung cancer (20, 23, 25, 28).

**Table 2:** Summary of included studies that evaluate the epithelial–mesenchymal transition and immune cell markers with tumor budding in lung cancer.

Sr. no.	Author/year/reference	EMT and other molecular features	Main findings
1	Yamaguchi, 2010 (23)	β-catenin, E-cadherin, laminin 5γ2, EGFR, IGF-1R, CAIX, GLUT-1, Vimentin, Surfactant protein-A, TTF-1, MMP-7, CD68, and CD204	Reduced expression of E-cadherin, β-catenin, and surfactant protein-A and increased expression of laminin 5**γ**2 in tumor budding cells (*p* < 0.005). No significant difference in CD44, growth factor receptor (EGFR and IGF-1R) hypoxia-induced protein (CAIX and GLUT-1), differentiation marker (TTF-1), MMP-7, and tumor-infiltrating macrophages (CD68 and CD204) between budding cells and near-budding cells.
2	Taira, 2011 (20)	EMT markers and other markers	E-cadherin (*p* = 0.004) and β-catenin (*p* = 0.002) levels in the tumor budding were significantly lower than solid nests. Laminin-5γ2 expression level in the tumor budding was significantly higher than solid nests (*p* = 0.001). Geminin-positive cells found more frequently in the TB cells than solid nests (median: 15 vs. 29; *p* = 0.008).
3	Kadota (2), 2015 (17)	Infiltrative immune-cell markers KRAS and EGFR	High-grade TB was significantly associated with high stromal CD3+ lymphocyte infiltration (*p* < 0.001), high stromal FoxP3+ lymphocyte infiltration (*p* < 0.001), high stromal FoxP3/CD3 risk index (*p* < 0.001), tumoral and stromal CD68 macrophage infiltration (*p* < 0.001), and tumoral IL-7R overexpression (*p* < 0.001). High-grade tumor budding was more frequently identified in KRAS wild type tumors than mutated tumors (*p* = 0.038). Tumor budding was not significantly associated with EGFR mutation.
4	Kadota (3), 2017 (25)	EMT markers	Increased expression of vimentin in high-grade compared to low-grade tumor budding (*p* = 0.023). Lower expression of E-cadherin was observed in the high-grade tumor budding in contrast to low-grade tumor budding (*p* = 0.003).
5.	Ammour, 2017 (28)	EMT markers	Reduced expression of E-cadherin and a significant increase in nuclear ZEB1.

EMT, epithelial–mesenchymal transition.

Furthermore, regarding immune cell infiltration, high stromal CD3^+^ lymphocytes, FoxP3^+^ lymphocytes, and CD68^+^ tumor and stromal infiltrating macrophages in TB were higher (**Table 2**) (17).

### Publication Bias

We used a funnel plot, Begg’s test, and Egger’s test to investigate publication bias. The funnel plot was asymmetric (**Supplementary Figure 7A**), and the trim-and-fill method was used to make the funnel plot symmetric (**Supplementary Figure 7B**). Furthermore, according to Begg’s test, no publication bias was found. However, Egger’s linear regression test identified publication bias in OS (*p* = 0.013) and tumor stage (*p* = 0.008) (**Supplementary Table 6**).

## Discussion

Our study demonstrated that high-grade TB was significantly correlated with poor prognosis (**Figures 1**, **2** and **Supplementary Figures 2, 3**). We also found that high-grade TB was associated with aggressive clinicopathological parameters and smoking history (**Supplementary Table 5** and **Supplementary Figures 4–6**). To the best of our knowledge, this is the first comprehensive systematic review and meta-analysis to evaluate the correlation between TB expression and lung cancer prognosis based on all available data pooled.

Although the development of effective therapeutic approaches has significantly improved the clinical outcome (3, 4), the survival rate of curatively resected lung cancer is still low (1, 4). High-risk groups should be selected for better clinical outcomes. Moreover, adjuvant chemotherapy for lung cancer is limited to patients with lung cancer beyond stage I. However, in a previous study, 30% of patients at those stages showed disease recurrence (6, 32). Actually, clinicians have some difficulty deciding on chemotherapy to prevent recurrence in stage I patients (6). We suggest that TB may help identify patients at high risk of recurrence and offer them adjuvant chemotherapy, especially in cases where they otherwise may not receive necessary treatment.

We found that high-grade TB was a poor prognostic factor in both LADC and LSCC with relatively low heterogeneity (**Figure 3**), regardless of ethnicity (**Figure 3B**). Originally, TB was recognized as a prognostic marker in colon adenocarcinoma (33, 34) and may be related to LADC due to the same histological subtype. Through our systematic analysis, we also confirmed the poor prognosis of high-grade TB in both LADC and LSCC. Hence, other histological subtypes of lung cancers, such as small cell lung carcinoma, large cell endocrine carcinoma, and adenosquamous carcinoma, remain a topic of discussion for research.

In this meta-analysis, three assessment methods (TB-YN, TB-1HPF, and TB-10HPF) were used to interpret TB on histological slides. Although the total heterogeneity was moderate (56%) in the total scoring system, all assessment methods showed poor OS with higher TB. Moreover, subgroup analysis showed that TB-YN (*I* ^2^ = 15%) criteria were more reproducible than TB-1HPF (*I* ^2^ = 56%) (**Figure 2A**). This might be because the TB-YN method is simple and more uniform than the TB-1HPF method. However, considering that TB imitators, like macrophages, tangentially sectioned tumor glands, or apoptotic tumor cells, can be interpreted as high-grade TB using the TB-YN method, dividing TB as present or absent could still result in an error (6, 10, 12). Therefore, standardization of the TB assessment method is urgently needed to predict a precise prognosis.

Ammour et al. revealed that the total number per tumor cluster was closely related to the EMT process. They found that a series of sequential events for EMT occurred at several different cell numbers per cluster (28). The current TB assessment method uses <5 tumor cells; however, this cutoff was not fully validated according to molecular markers (28). Therefore, the cutoff point for TB definition can be improved by further studies.

Recently, ITBCC has been known as a very popular scoring system among pathologists, which was approved in 2016 to create a standardized scoring system for colorectal cancer (8) and was further validated in 2019 (35), and is currently recognized as an independent prognostic marker (8). Regardless of the organ-specific scoring system, pathologists are currently using this scoring system for other cancers (36, 37) including lung cancer (19). For instance, Neppl et al. validated the five-step ITBCC guidelines for 354 LSCC cases and found it to be a significant independent prognostic parameter for OS (HR 1.51, 95% CI 1.186–2.108, *p* = 0.002) (19). Such a scoring system affiliated with the reputed committee still needs to be standardized for lung cancer pathology reports.

Moreover, there is a discussion among pathologists regarding whether H&E or immunohistochemical staining with pan-cytokeratin (AE1/AE3) antibody is better for TB scoring. The major advantage of the AE1/AE3 antibody is that it shows TBs more clearly and reduces the subjectivity variation during the examination of slide (7). However, it also stained apoptotic cells and other cell-related debris, which should not be counted in the final number (8). Moreover, emerging evidence in lung cancer showed that there was no significant difference between staining and gave an equal result (*R* = 0.92, *p* < 0.001) (19). Similarly, previous meta-analyses conducted on colon cancer showed a similar prognostic value for TB using both H&E and immunohistochemistry (38). Moreover, ITBCC suggested that H&E staining should be used for routine diagnosis because of the cheaper price, while AE1/AE3 should be exploited for complicated cases (8). Future studies or consensus meetings are still required to scrutinize the differences between these two types of staining.

Intriguingly, smoking was one of the most important findings associated with the higher TB observed by our meta-analysis. To the best of our knowledge, this is the first study that revealed the association of smoking with high-grade TB. Previously, a few articles tried to reveal the association of high-grade TB and smoking; however, all of them could not show a significant relationship, which may be due to the inadequate sample size (**Supplementary Table 6**) (17, 20, 23).

This may generate a hypothesis that the association between TB and smoking may be linked to the EMT process (39). In the EMT process, epithelial cells lose their epithelial appearance, marked by the reduced expression of E-cadherin. They then acquire the spindle shape of the cell, marked by the increased expression of vimentin, Twist1, and Snail2. This is known as a mesenchymal transition (39–41). Previously, Zhao et al. treated a lung cancer cell line (A549) with a cigarette smoking extract that activated the EMT process *via* the NF-κB pathway (41). Subsequently, they found that increased expression of IL-6, N-cadherin, and vimentin leads to malignant transformation of cells (41). Similarly, mesenchymal markers were increased *via* the WNT3a/β-catenin pathway when human bronchial epithelial cells were exposed to nicotine (42). These results suggest that there may be a high possibility of TB in smokers, which may be activated through the EMT pathway.

Since the last decade, many researchers have investigated the relationship between TB and EMT in various cancers, such as pancreatic ductal adenocarcinoma (43) and colorectal cancer (44). Through our systematic analysis, we found four studies that showed a significant relationship between EMT and high-grade TB. Decreased cell adhesion molecule (E-cadherin), WNT signaling activation (decreased β-catenin), mesenchymal protein expression (vimentin), invasiveness or cell migration (laminin-5γ2), increased EMT transcription protein (ZEB1), and decreased proliferative index (geminin) were observed in lung cancer TB cells (20). Interestingly, high-grade TB was more observed in KRAS wild-type LSCC (17, 34, 45). KRAS mutation is known as a promotor for EMT process in colorectal carcinoma (34, 45); however, KRAS mutation in lung cancer is only known to be related to high mutation burden and PD-L1 expression (46). Further studies about KRAS mutation in lung cancer for TB and/or EMT are still needed.

Interestingly, Ammour et al. revealed that TB may or may not be connected to the main mass by using three-dimensional reconstruction (28). However, connection to the main mass of TB or the lack of it was not important; instead, the total cell number per tumor clustered was significantly associated with the EMT process. Also, they found that E-cadherin was the first event of the EMT process and the E-cadherin/ZEB1 axis played a crucial role in the change of cellular morphology (28). Overall, these results suggest that TB involves various morphological changes similar to EMT that transform the tumor cell into a more invasive and aggressive form.

Furthermore, there has been an association between TB and the TME that helps in the progression of tumor (7, 47). In this regard, we also found two studies that used tumor infiltrate immune cells. For instance, one study from the USA showed that protumor immune cells present in high-grade TB (17), while another study from Japan did not show significant results (18). The conflicting results in the same histological subtype (adenocarcinoma) may be due to ethnicity, study design, and sample size. Further studies are required to validate this hypothesis.

There are some limitations to the present meta-analysis that should be addressed here. First, in the absence of an HR, we extracted the data through the Kaplan–Meier curve, which may be less accurate than data directly obtained from articles for the purpose of collecting all available data. Second, research conducted on Asian people and LADC was relatively less. Third, the number of studies included in this meta-analysis is limited; therefore, further studies on the prognosis of TB in lung cancer are still needed, especially beyond LADC and LSCC, and Asian people.

## Conclusion

Our study concludes that high-grade TB is significantly associated with poor prognosis and aggressive clinicopathological features regardless of histologic type and ethnicity. Although various kinds of assessment methods also showed similar results, in real practice, standardization for assessment methods by large consensus meetings is still needed. Moreover, EMT and smoking revealed a significant relationship with high-grade tumors. We believe that TB should be implemented routinely when reporting pathological diagnoses.

## Data Availability Statement

The original contributions presented in the study are included in the article/**Supplementary Material**. Further inquiries can be directed to the corresponding author.

## Author Contributions

Conceptualization: NT and KY. Data curation: NT, YC, and KY. Formal analysis: NT and MA. Funding acquisition: KY. Investigation: NT and KY. Project administration: OS, YC, and KY. Supervision: YC and KY. Validation: NT, YC, and KY. Visualization: NT and MA. Writing original draft: NT and KY. Review and editing: NT, MA, YC, OS, and KY. All authors contributed to the article and approved the submitted version.

## Funding

This research was supported by the Basic Science Research Program through the National Research Foundation of Korea (NRF) funded by the Ministry of Education (2021R1I1A1A01060037). The authors wish to acknowledge the financial support of the Catholic University of Korea, Uijeongbu St. Mary’s Hospital Clinical Research Laboratory Foundation made in the program year of 2021 (UJBCRL202125).

## Conflict of Interest

The authors declare that the research was conducted in the absence of any commercial or financial relationships that could be construed as a potential conflict of interest.

## Publisher’s Note

All claims expressed in this article are solely those of the authors and do not necessarily represent those of their affiliated organizations, or those of the publisher, the editors and the reviewers. Any product that may be evaluated in this article, or claim that may be made by its manufacturer, is not guaranteed or endorsed by the publisher.
