# Supplementary material for: Tumor Budding as a Marker for Poor Prognosis and Epithelial–Mesenchymal Transition in Lung Cancer: A Systematic Review and Meta-Analysis
Source: Front Oncol. 2022 Jun 2;12:828999. doi: 10.3389/fonc.2022.828999 (PMC9201279; doi:10.3389/fonc.2022.828999)
Supplement: Supplementary file 1 [file DataSheet_1.docx]

**Tumor Budding as a Marker for Poor Prognosis and Epithelial-Mesenchymal Transition in Lung Cancer: A Systematic Review and Meta-Analysis**

**Supplementary Data**

**
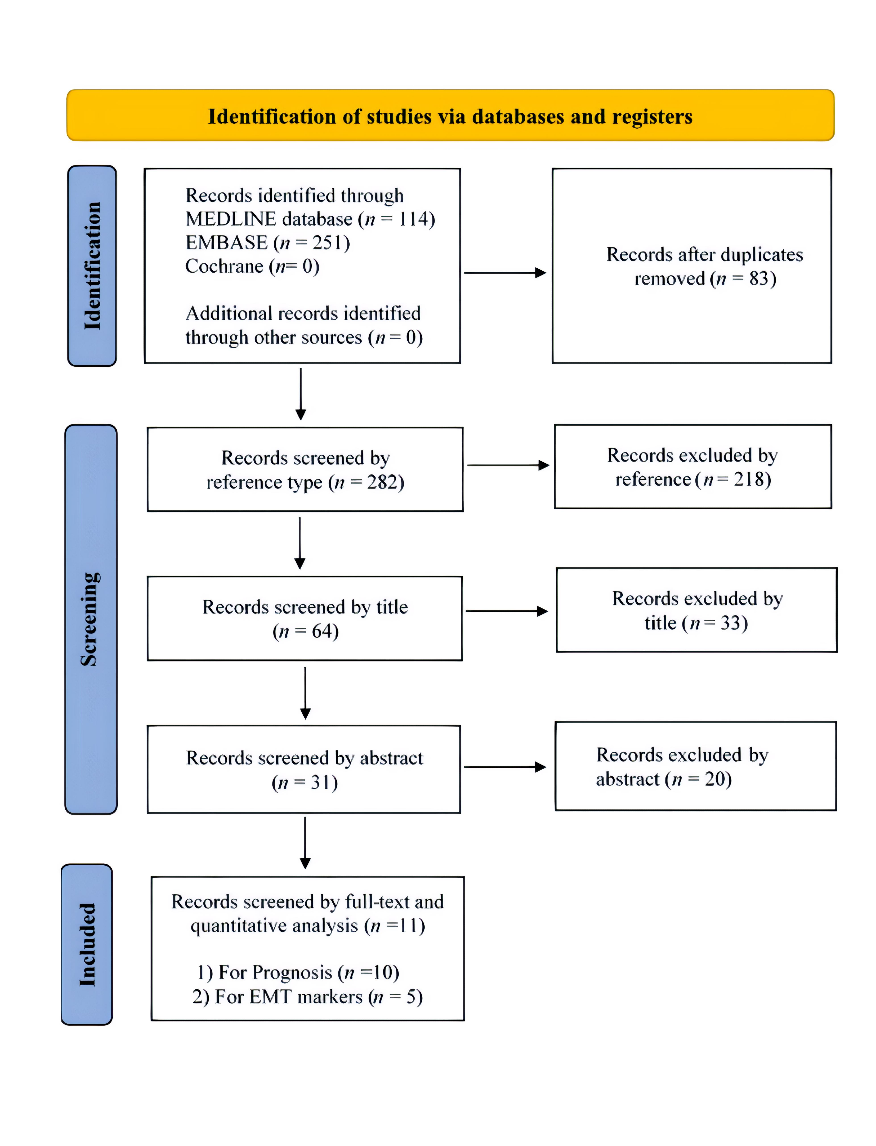
**

**Supplementary Figure 1.** **PRISMA flow diagram showing the study selection process.**

| 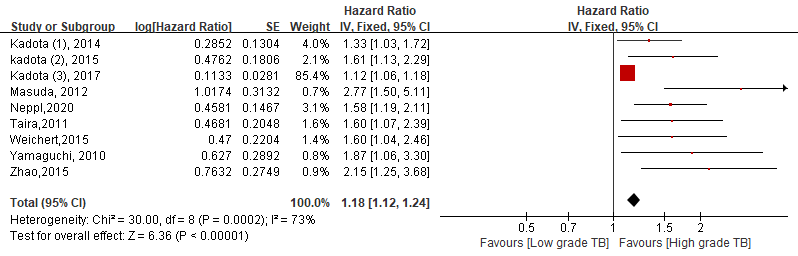   1. Overall survival | 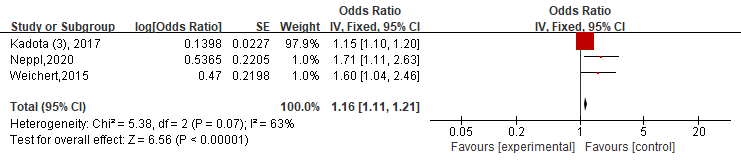   1. Disease-free survival |
| --- | --- |

**Supplementary Figure 2. Forest plot evaluating the (A) overall survival and (B) disease free survival of all included studies.**

| 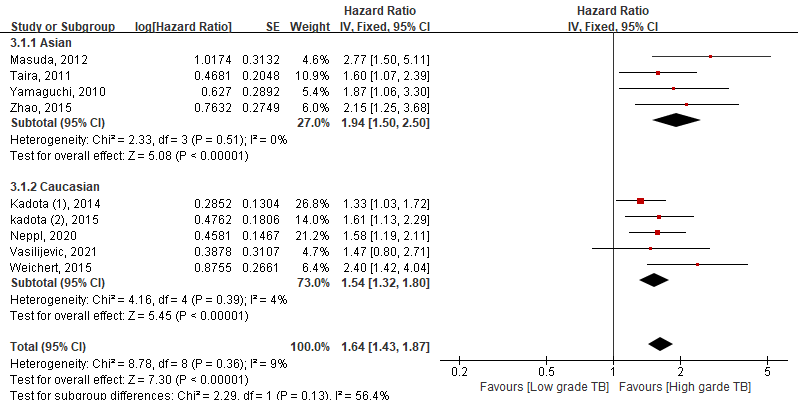  A (Ethnicity type) | 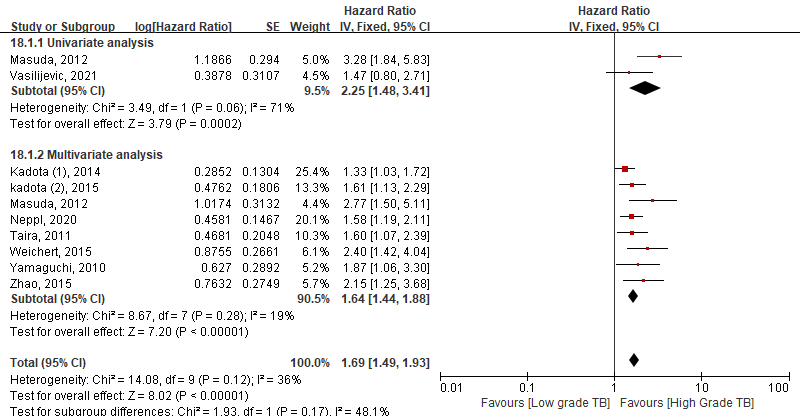  B (Univariate & Multivariate analysis) |
| --- | --- |

**Supplementary Figure 3. Subgroup hazard ratios analyze the tumor budding expression and overall survival by ethnicity (A) and by univariate vs multivariate analysis (B) in lung cancer patients.**

**
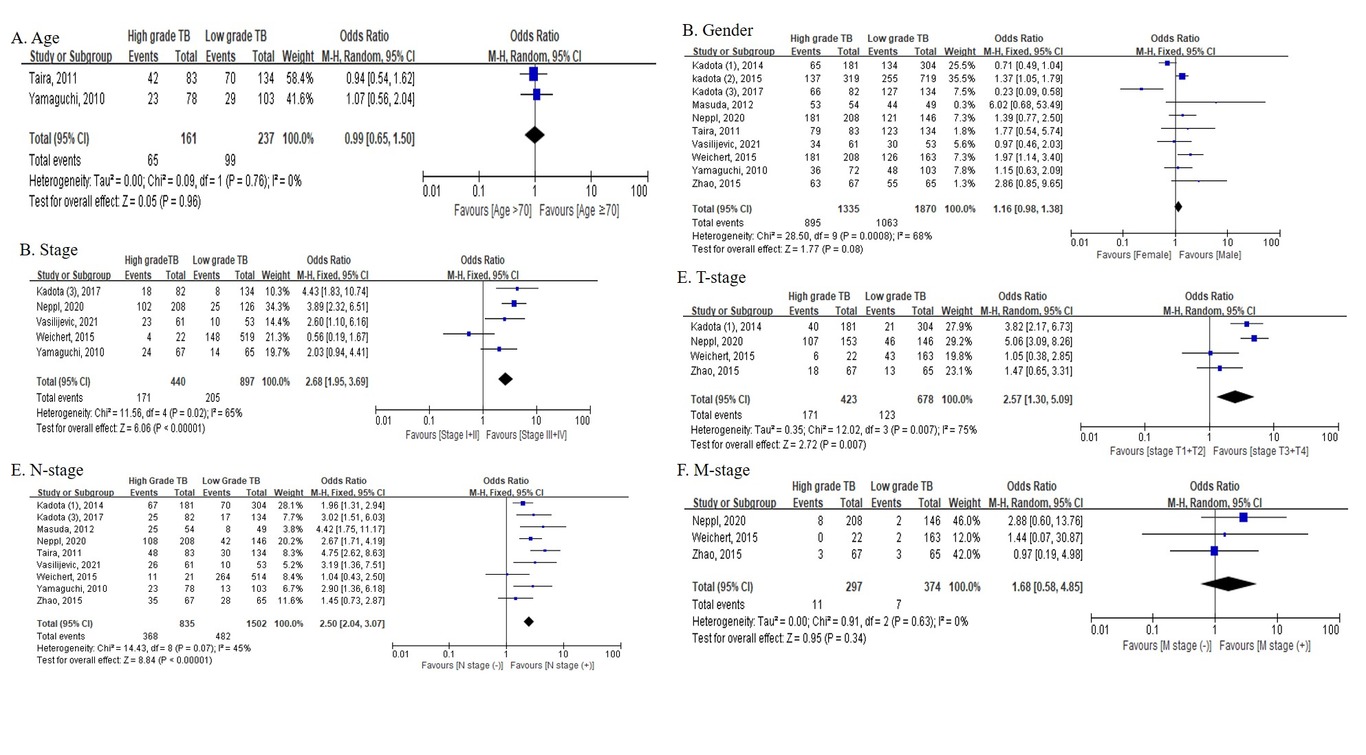
**

**Supplementary Figure 4. Subgroup hazard ratio analyzing evaluating the tumor budding expression and clinical parameters such as (A) age, (B) gender, (C) stage, (D) T-stage, (E) N stage, (F) M stage in lung cancer patients.**

**
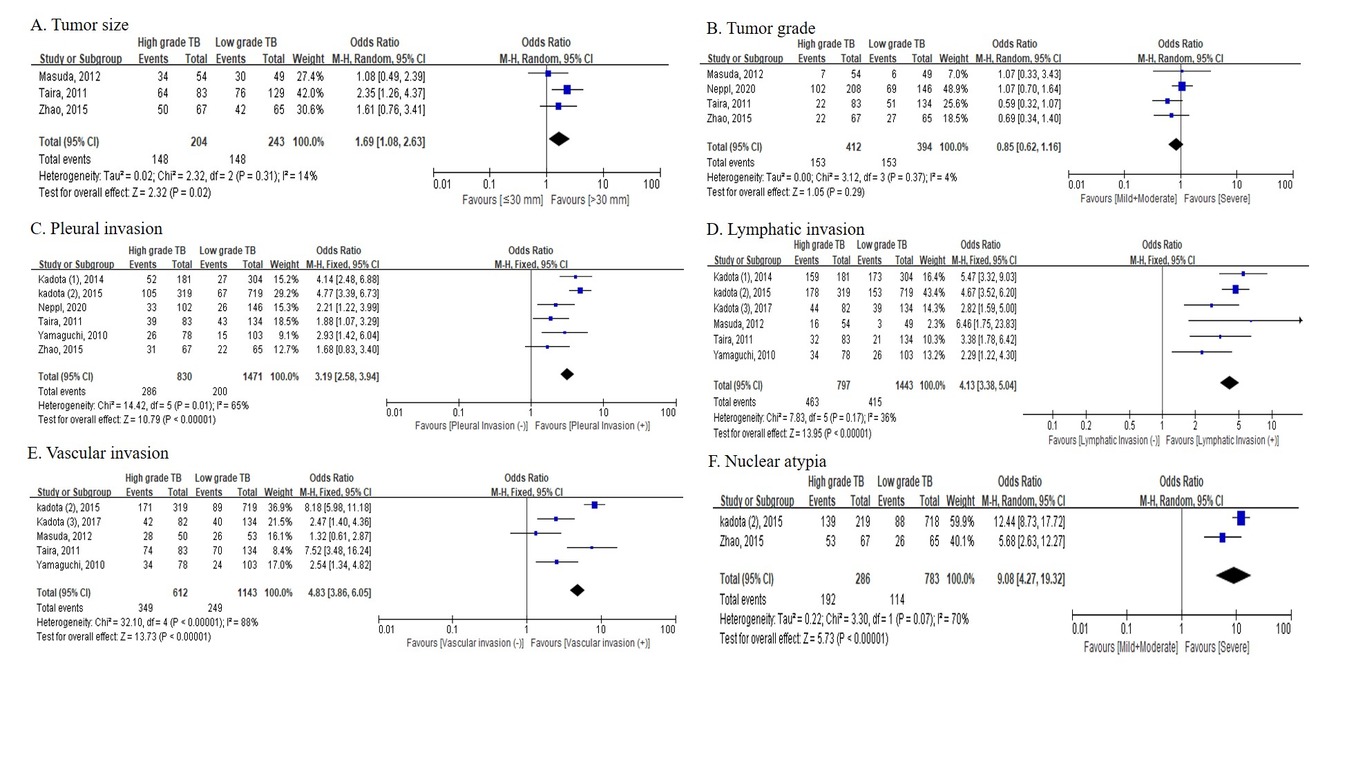
**

**Supplementary Figure 5. Subgroup hazard ratio analyzing the tumor budding expression and pathological parameters in lung cancer patients (A) tumor size, (B) tumor grade, (C) pleural invasion, (D) lymphatic invasion, (E) vascular invasion, (F) nuclear atypia**


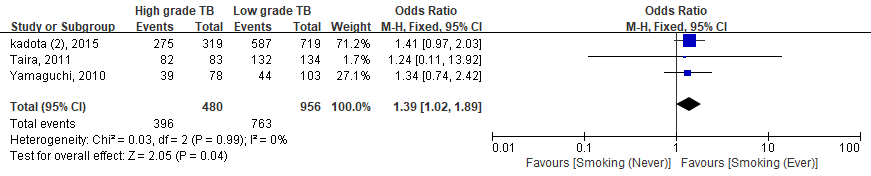


**Supplementary Figure 6. Subgroup hazard ratio analyzing the tumor budding expression and smoking in lung cancer patients.**

| 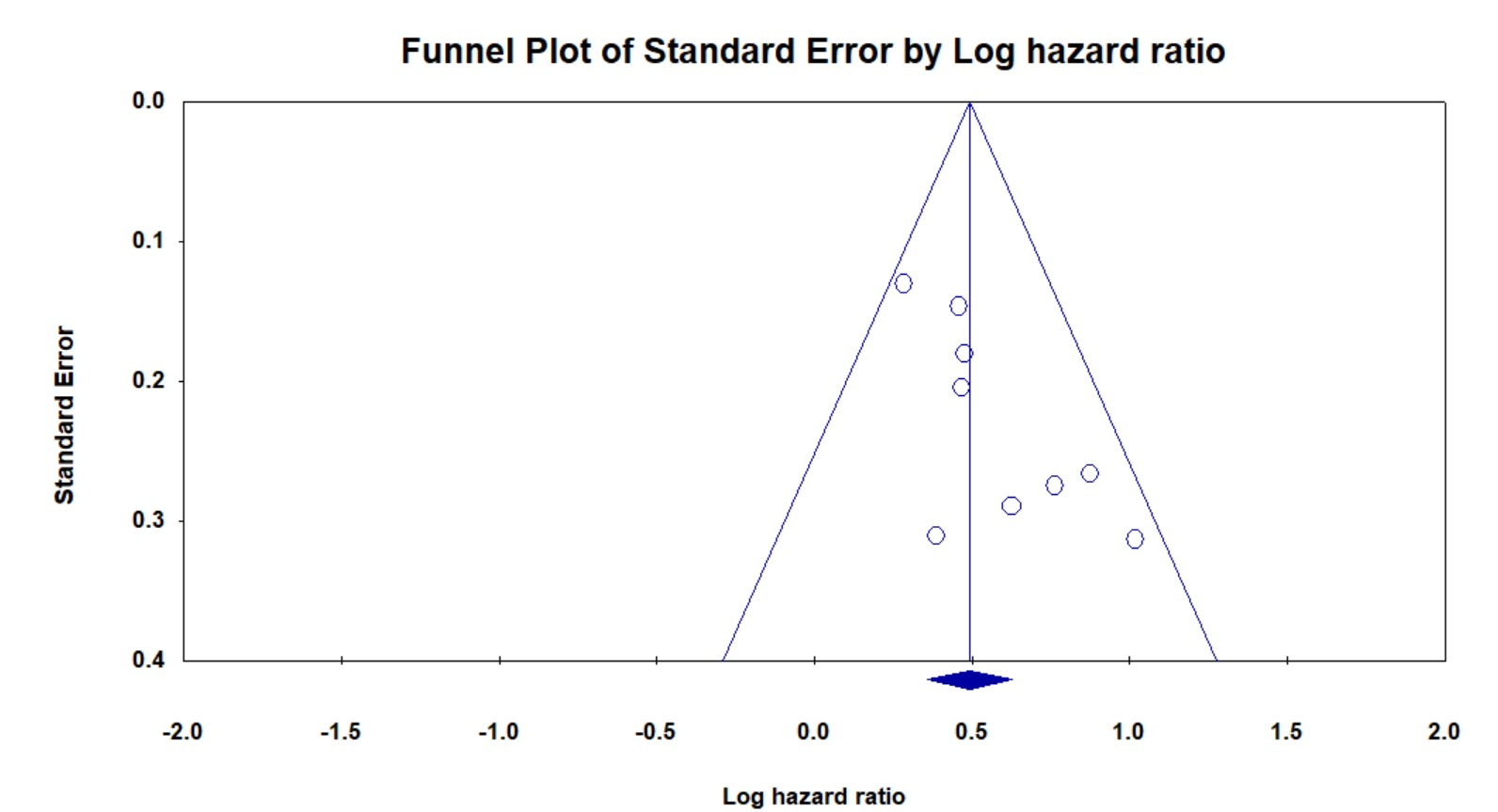  **A. Funnel plot** | 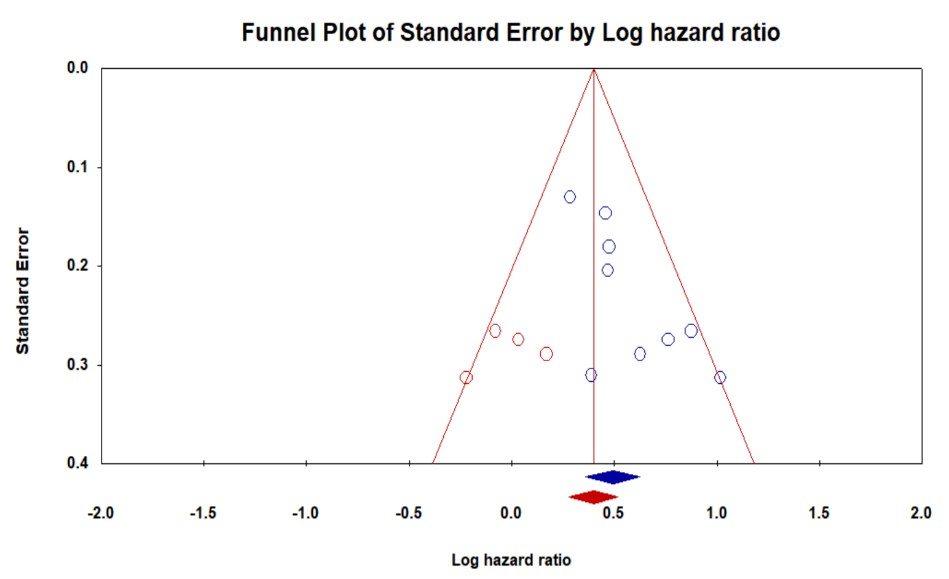  **B. Trim and fill method** |
| --- | --- |

**Supplementary Figure 7. (A) Funnel plot and (B) trim and fill method showing the relationship between tumor budding and overall survival.**

**Supplementary Table 1. Detailed overview of key-words used for search strategy**

| **Database** | **Search** | **Keywords** |
| --- | --- | --- |
| **MEDLINE** | #1 | "Lung Neoplasms"[Mesh] |
|  | #2 | "Lung Neoplasms"[TW] OR "Pulmonary Neoplasms"[TW] OR "Neoplasms, Lung"[TW] OR "Lung Neoplasm"[TW] OR "Neoplasm, Lung"[TW] OR "Neoplasms, Pulmonary"[TW] OR "Neoplasm, Pulmonary"[TW] OR "Pulmonary Neoplasm"[TW] OR "Lung Cancer"[TW] OR "Cancer, Lung"[TW] OR "Cancers, Lung"[TW] OR "Lung Cancers"[TW] OR "Pulmonary Cancer"[TW] OR "Cancer, Pulmonary"[TW] OR "Cancers, Pulmonary"[TW] OR "Pulmonary Cancers"[TW] OR "Cancer of the Lung"[TW] OR "Cancer of Lung"[TW] OR "lung carcinoma"[TW] |
|  | #3 Combine | #1 OR #2 |
|  | #4 | "Lung"[Mesh] OR "Lung"[TW] OR "Lungs"[TW] |
|  | #5 | "Carcinoma"[Mesh] |
|  | #6 | "Carcinoma"[TW] OR "Carcinomas"[TW] OR "Epithelial Neoplasms, Malignant"[TW] OR "Malignant Epithelial Neoplasms"[TW] OR "Epithelial Neoplasm, Malignant"[TW] OR "Malignant Epithelial Neoplasm"[TW] OR "Neoplasm, Malignant Epithelial"[TW] OR "Neoplasms, Malignant Epithelial"[TW] OR "Epithelial Tumors, Malignant"[TW] OR "Epithelial Tumor, Malignant"[TW] OR "Malignant Epithelial Tumor"[TW] OR "Malignant Epithelial Tumors"[TW] OR "Tumor, Malignant Epithelial"[TW] OR "Epithelioma"[TW] OR "Epitheliomas"[TW] OR "Carcinoma, Undifferentiated"[TW] OR "Undifferentiated Carcinoma"[TW] OR "Undifferentiated Carcinomas"[TW] OR "Carcinoma, Anaplastic"[TW] OR "Anaplastic Carcinoma"[TW] OR "Anaplastic Carcinomas"[TW] OR "Carcinoma, Spindle-Cell"[TW] OR "Carcinoma, Spindle Cell"[TW] OR "Spindle-Cell Carcinoma"[TW] OR "Spindle-Cell Carcinomas"[TW] OR "Carcinomatosis"[TW] OR "Carcinomatoses"[TW] OR "solid carcinoma"[TW] |
|  | #7 Combine | #5 OR #6 |
|  | #8  Combine | #3 OR (#4 AND #7) |
|  | #9 Combine | "tumor budding"[TW] OR "budding"[TW] OR "high grade tumor budding"[TW] OR "tumor cell dissociation"[TW] OR "single tumor cell"[TW] OR "individual tumor cell"[TW] OR "small groups of tumor cell"[TW] |
|  | #10 Combine | #8 AND #9 |

| **Data base** | **Search** | **Keywords** |
| --- | --- | --- |
| **EMBASE** | #1 | "lung tumor"/exp |
|  | #2 | "Lung Neoplasms":ti,ab,kw,de OR "Pulmonary Neoplasms":ti,ab,kw,de OR "Neoplasms, Lung":ti,ab,kw,de OR "Lung Neoplasm":ti,ab,kw,de OR "Neoplasm, Lung":ti,ab,kw,de OR "Neoplasms, Pulmonary":ti,ab,kw,de OR "Neoplasm, Pulmonary":ti,ab,kw,de OR "Pulmonary Neoplasm":ti,ab,kw,de OR "Lung Cancer":ti,ab,kw,de OR "Cancer, Lung":ti,ab,kw,de OR "Cancers, Lung":ti,ab,kw,de OR "Lung Cancers":ti,ab,kw,de OR "Pulmonary Cancer":ti,ab,kw,de OR "Cancer, Pulmonary":ti,ab,kw,de OR "Cancers, Pulmonary":ti,ab,kw,de OR "Pulmonary Cancers":ti,ab,kw,de OR "Cancer of the Lung":ti,ab,kw,de OR "Cancer of Lung":ti,ab,kw,de OR "lung carcinoma":ti,ab,kw,de |
|  | #3 Combine | #1 OR #2 |
|  | #4 | "lung"/exp OR "Lung":ti,ab,kw,de OR "Lungs":ti,ab,kw,de |
|  | #5 | "carcinoma"/exp |
|  | #6 | "Carcinoma":ti,ab,kw,de OR "Carcinomas":ti,ab,kw,de OR "Epithelial Neoplasms, Malignant":ti,ab,kw,de OR "Malignant Epithelial Neoplasms":ti,ab,kw,de OR "Epithelial Neoplasm, Malignant":ti,ab,kw,de OR "Malignant Epithelial Neoplasm":ti,ab,kw,de OR "Neoplasm, Malignant Epithelial":ti,ab,kw,de OR "Neoplasms, Malignant Epithelial":ti,ab,kw,de OR "Epithelial Tumors, Malignant":ti,ab,kw,de OR "Epithelial Tumor, Malignant":ti,ab,kw,de OR "Malignant Epithelial Tumor":ti,ab,kw,de OR "Malignant Epithelial Tumors":ti,ab,kw,de OR "Tumor, Malignant Epithelial":ti,ab,kw,de OR "Epithelioma":ti,ab,kw,de OR "Epitheliomas":ti,ab,kw,de OR "Carcinoma, Undifferentiated":ti,ab,kw,de OR "Undifferentiated Carcinoma":ti,ab,kw,de OR "Undifferentiated Carcinomas":ti,ab,kw,de OR "Carcinoma, Anaplastic":ti,ab,kw,de OR "Anaplastic Carcinoma":ti,ab,kw,de OR "Anaplastic Carcinomas":ti,ab,kw,de OR "Carcinoma, Spindle-Cell":ti,ab,kw,de OR "Carcinoma, Spindle Cell":ti,ab,kw,de OR "Spindle-Cell Carcinoma":ti,ab,kw,de OR "Spindle-Cell Carcinomas":ti,ab,kw,de OR "Carcinomatosis":ti,ab,kw,de OR "Carcinomatoses":ti,ab,kw,de OR "solid carcinoma":ti,ab,kw,de |
|  | #7 Combine | #5 OR #6 |
|  | #8  Combine | #3 OR (#4 AND #7) |
|  | #9 Combine | "tumor budding":ti,ab,kw,de OR "budding":ti,ab,kw,de OR "high grade tumor budding":ti,ab,kw,de OR "tumor cell dissociation":ti,ab,kw,de OR "single tumor cell":ti,ab,kw,de OR "individual tumor cell":ti,ab,kw,de OR "small groups of tumor cell":ti,ab,kw,de |
|  | #10 Combine | #8 AND #9 |

| **Data base** | **Search** | **Keywords** |
| --- | --- | --- |
| **Cochrane Library** | #1 | [mh "Lung Neoplasms"] |
|  | #2 | "Lung Neoplasms":ti,ab,kw OR "Pulmonary Neoplasms":ti,ab,kw OR "Neoplasms, Lung":ti,ab,kw OR "Lung Neoplasm":ti,ab,kw OR "Neoplasm, Lung":ti,ab,kw OR "Neoplasms, Pulmonary":ti,ab,kw OR "Neoplasm, Pulmonary":ti,ab,kw OR "Pulmonary Neoplasm":ti,ab,kw OR "Lung Cancer":ti,ab,kw OR "Cancer, Lung":ti,ab,kw OR "Cancers, Lung":ti,ab,kw OR "Lung Cancers":ti,ab,kw OR "Pulmonary Cancer":ti,ab,kw OR "Cancer, Pulmonary":ti,ab,kw OR "Cancers, Pulmonary":ti,ab,kw OR "Pulmonary Cancers":ti,ab,kw OR "Cancer of the Lung":ti,ab,kw OR "Cancer of Lung":ti,ab,kw OR "lung carcinoma":ti,ab,kw |
|  | #3 Combine | #1 OR #2 |
|  | #4 | [mh "Lung"] OR "Lung":ti,ab,kw OR "Lungs":ti,ab,kw |
|  | #5 | [mh "Carcinoma"] |
|  | #6 | "Carcinoma":ti,ab,kw OR "Carcinomas":ti,ab,kw OR "Epithelial Neoplasms, Malignant":ti,ab,kw OR "Malignant Epithelial Neoplasms":ti,ab,kw OR "Epithelial Neoplasm, Malignant":ti,ab,kw OR "Malignant Epithelial Neoplasm":ti,ab,kw OR "Neoplasm, Malignant Epithelial":ti,ab,kw OR "Neoplasms, Malignant Epithelial":ti,ab,kw OR "Epithelial Tumors, Malignant":ti,ab,kw OR "Epithelial Tumor, Malignant":ti,ab,kw OR "Malignant Epithelial Tumor":ti,ab,kw OR "Malignant Epithelial Tumors":ti,ab,kw OR "Tumor, Malignant Epithelial":ti,ab,kw OR "Epithelioma":ti,ab,kw OR "Epitheliomas":ti,ab,kw OR "Carcinoma, Undifferentiated":ti,ab,kw OR "Undifferentiated Carcinoma":ti,ab,kw OR "Undifferentiated Carcinomas":ti,ab,kw OR "Carcinoma, Anaplastic":ti,ab,kw OR "Anaplastic Carcinoma":ti,ab,kw OR "Anaplastic Carcinomas":ti,ab,kw OR "Carcinoma, Spindle-Cell":ti,ab,kw OR "Carcinoma, Spindle Cell":ti,ab,kw OR "Spindle-Cell Carcinoma":ti,ab,kw OR "Spindle-Cell Carcinomas":ti,ab,kw OR "Carcinomatosis":ti,ab,kw OR "Carcinomatoses":ti,ab,kw OR "solid carcinoma":ti,ab,kw |
|  | #7 Combine | #5 OR #6 |
|  | #8  Combine | #3 OR (#4 AND #7) |
|  | #9 Combine | "tumor budding":ti,ab,kw OR "budding":ti,ab,kw OR "high grade tumor budding":ti,ab,kw OR "tumor cell dissociation":ti,ab,kw OR "single tumor cell":ti,ab,kw OR "individual tumor cell":ti,ab,kw OR "small groups of tumor cell":ti,ab,kw |
|  | #10 Combine | #8 AND #9 |

**Supplementary Table 2. New Castle Ottawa scoring system**

| **First author, year** | **Selection^1^** | | | | **Comparability^2^** | **Outcome^3^** | | |  |
| --- | --- | --- | --- | --- | --- | --- | --- | --- | --- |
|  | **Exposed Cohort ★** | **non-exposed cohort ★** | **Ascertainment of exposure ★** | **Outcome of the study was not presented at the start of study★** | **Control for important factor or additional factor ★★** | **Outcome**  **Assessment ★** | **Adequate**  **follow-up★** | **Loss to follow-up★** | **Total Score** |
| Taira 2011(1) | ★ | ★ | ★ |  | ★★ | ★ | ★ | ★ | 8 |
| Masuda,2012(2) | ★ | ★ | ★ |  | ★★ | ★ | ★ | ★ | 8 |
| Kadota (1) 2014(3) | ★ | ★ | ★ |  | ★★ | ★ | ★ | ★ | 8 |
| Weichert 2015(4) | ★ | ★ | ★ |  | ★★ | ★ | ★ | ★ | 8 |
| Zhao 2015(5) | ★ | ★ | ★ |  | ★★ | ★ | ★ |  | 7 |
| Kadota (3)(6) | ★ | ★ | ★ |  | ★ | ★ | ★ | ★ | 7 |
| Neppl 2020(7) | ★ | ★ | ★ |  | ★★ | ★ | ★ |  | 7 |
| Yamaguchi 2010(8) | ★ | ★ | ★ |  | ★★ | ★ | ★ | ★ | 8 |
| Kadota (2) 2015(9) | ★ | ★ | ★ |  | ★★ | ★ | ★ | ★ | 8 |
| Ammour, 2107(10) | ★ | - | ★ |  | - | - | - | - | 2 |
| Vasilijević 2021(11) | ★ | ★ | ★ |  | ★★ | ★ | ★ |  | 7 |

1 “Selection” part includes representativeness of cases, selection of controls, exposure ascertainment, and no death when the investigation started.

2 “Comparability” part includes a comparison between each group and addition factors comparable on confounders.

3 “Outcome” part includes outcome assessment, adequate follow-up, and loss to follow-up rate.

**Supplementary Table 3. Main Characteristics of all lung cancer studies included studies**

| Histological subtype | Authors | Year | Country | Age (yrs) | Stage | Enrollment period | Pathologist  Number | Follow up (yrs) | Treatment  (n) | HR (95% CI) | NOS  Score |
| --- | --- | --- | --- | --- | --- | --- | --- | --- | --- | --- | --- |
| LSCC | Taira (1) | 2011 | Japan | 44-88 | I-III | 2000-2010 | 2 | 4.7 | No treatment | OS: 1.597 (1.069-2.384) | 8 |
|  | Masuda (2) | 2012 | Japan | -  (43-85) | - | NA | - | 4.2 | - | OS: 2.766 (1.497-5.109) | 8 |
|  | Kadota (1) (3) | 2014 | USA | 71  (39-88) | I-III | 1999-2009 | 2 | 4.1 | - | OS: 1.33 (1.03-1.70) | 8 |
|  | Weichert (4) | 2015 | Germany | Mean 64.8  (38-82) | I-IV | 2002-2010 | 2 | 3.1 | Adjuvant.  chemotherapy (112) | OS:2.40 (1.42-4.04)  DFS: 1.60 (1.04-2.46) | 8 |
|  | Zhao (5) | 2015 | China | 56  (38-80) | I-IV | 2007-2008 | - | - | - | OS: 0.466 (0.272-0.799) | 7 |
|  | Kadota (3) (6) | 2017 | Japan | 74  (41-89) | I-IV | 1999-2012 | 1 | 4.1 | Adjuvant.  chemotherapy (35 ) | DFS: 1.15 (1.10-1.21) | 7 |
|  | Neppl (7) | 2020 | Switzerland | 69.09  (43-85) | I-IV | 2000-2013 | - | - | Adjuvant.  chemotherapy (75) | OS: 1.581(1.186-2.108)  DFS: 1.710(1.11-2.632)  PFS: 1.457(1.123-1.89) | 7 |
| LADC | Yamaguchi (8) | 2010 | Japan | 62  (33-84) | I-III | 1993-1998 | 2 | 10.0 | - | OS: 1.872 (1.062-3.298) | 8 |
|  | Kadota (2) (9) | 2015 | USA | 69  (23-96) | IA-IB | 1995-2009 | 2 | 3.1 | Adjuvant.  chemotherapy (14) | OS: 1.61 (1.13-2.29) | 8 |
|  | Ammour (10)* | 2017 | Germany | - | - | - | - | - | - | - | 2 |
|  | Vasilijević (11) | 2021 | Serbia | 63  (46-78) | I-III | 2018 | 2 | - | No treatment | OS: 1.47 (0.80-2.71) | 7 |

**Abbreviation:** EMT: epithelial and mesenchymal transition, NOS: Newcastle Ottawa score system, LADC: lung adenocarcinoma; LSCC: lung squamous cell carcinoma, Mag.: magnification, TB: tumor budding, OS: overall survival, DFS: disease-free survival, PFS: progression-free survival, RFS: recurrence-free survival, H&E: hematoxylin and eosin, IHC: Immunohistochemistry. ^*^In Ammour study contains pancreatic cancer, breast cancer, colorectal cancer, and lung cancers. (3 cases in each cancer)

**Supplementary Table 4. Characteristics of all studies clinicopathological data included in the final meta-analysis.**

| Clinicopathological parameters | | Kadota (1), 2014 (3) | | Kadota (2), 2015 (9) | | Kadota (3), 2017 (6) | | Masuda, 2012 (2) | | Neppl, 2019 (7) | | Taira 2012 (1) | | Vasilijević, 2021 (11) | | Weichert,2016  (4) | | Yamaguchi, 2010 (8) | | Zhao, 2015 (5) | |
| --- | --- | --- | --- | --- | --- | --- | --- | --- | --- | --- | --- | --- | --- | --- | --- | --- | --- | --- | --- | --- | --- |
|  |  | Low TB | High  TB | Low TB | High  TB | Low TB | High  TB | Low TB | High  TB | Low TB | High  TB | Low TB | High  TB | Low TB | High  TB | Low TB | High  TB | Low TB | High  TB | Low TB | High  TB |
| Age | <70 | **-** | **-** | **-** | **-** | **-** | **-** | **-** | **-** | **-** | **-** | 64 | 41 | - | - | - | - | 74 | 55 | **-** | **-** |
|  | ≥70 | **-** | **-** | **-** | **-** | **-** | **-** | **-** | **-** | **-** | **-** | 70 | 42 | - | - | - | - | 29 | 23 | **-** | **-** |
| Gender | Male | 134 | 65 | 255 | 137 | 127 | 66 | 44 | 53 | 121 | 181 | 123 | 79 | 34 | 30 | 423 | 19 | 48 | 36 | 55 | 63 |
|  | Female | 170 | 116 | 464 | 182 | 7 | 16 | 5 | 1 | 25 | 27 | 11 | 4 | 27 | 23 | 96 | 3 | 55 | 42 | 10 | 4 |
| Smoking | Never | - | - | 132 | 44 | - | - | - | - | - | - | 2 | 1 | - | - | - | - | 59 | 39 | - | - |
|  | Ever | - | - | 587 | 275 | - | - | - | - | - | - | 132 | 82 | - | - | - | - | 44 | 39 | - | - |
| Tumor Size (mm) | ≤30 mm | - | - | - | - | - | - | 19 | 20 | - | - | 58 | 19 | - | - | - | - | - | - | 23 | 17 |
|  | >30 mm | - | - | - | - | - | - | 30 | 34 | - | - | 76 | 64 | - | - | - | - | - | - | 42 | 50 |
| Tumor Grade | G1/G2 | - | - | - | - | - | - | 43 | 47 | 77 | 106 | 83 | 61 | - | - | - | - | - | - | 38 | 45 |
|  | G3 | - | - | - | - | - | - | 6 | 7 | 69 | 102 | 51 | 22 | - | - | - | - | - | - | 27 | 22 |
| T stage | T1+T2 | 283 | 141 | - | - | - | - | - | - | 100 | 101 | - | - | - | - | 414 | 16 | - | - | 52 | 49 |
|  | T3+T4 | 21 | 40 | - | - | - | - | - | - | 46 | 107 | - | - | - | - | 105 | 6 | - | - | 13 | 18 |
| N stage | Negative | 234 | 114 | - | - | 117 | 57 | 41 | 29 | 104 | 100 | 104 | 35 | 43 | 35 | 250 | 10 | 90 | 55 | 37 | 32 |
|  | Positive | 70 | 67 | - | - | 17 | 25 | 8 | 25 | 42 | 108 | 30 | 48 | 10 | 26 | 264 | 11 | 13 | 23 | 28 | 35 |
| M stage | Negative | - | - | - | - | - | - | - | - | 144 | 200 | - | - | - | - | 514 | 22 | - | - | 62 | 64 |
|  | Positive | - | - | - | - | - | - | - | - | 2 | 8 | - | - | - | - | 5 | 0 | - | - | 3 | 3 |
| Pathological stage | I+II | - | - | - | - | 126 | 64 | - | - | 121 | 106 | - | - | 43 | 38 | 371 | 18 | - | - | 51 | 43 |
|  | III+IV | - | - | - | - | 8 | 18 | - | - | 25 | 102 | - | - | 10 | 23 | 148 | 4 | - | - | 14 | 24 |
| Pleural Invasion | Negative | 277 | 129 | 652 | 214 | - | - | - | - | 120 | 140 | 91 | 44 | - | - | - | - | 88 | 52 | 43 | 36 |
|  | Positive | 27 | 52 | 67 | 105 | - | - | - | - | 26 | 63 | 43 | 39 | - | - | - | - | 15 | 26 | 22 | 31 |
| Lymphatic Invasion | Negative | - | - | 566 | 141 | 95 | 38 | 46 | 38 | - | - | 113 | 51 | - | - | - | - | 77 | 44 | - | - |
|  | Positive | - | - | 153 | 178 | 39 | 44 | 3 | 16 | - | - | 21 | 32 | - | - | - | - | 26 | 34 | - | - |
| Vascular Invasion | Negative | - | - | 630 | 148 | 94 | 40 | 27 | 26 | - | - | 64 | 9 | - | - | - | - | 79 | 44 | - | - |
|  | Positive | - | - | 89 | 171 | 40 | 42 | 22 | 28 | - | - | 70 | 74 | - | - | - | - | 24 | 34 | - | - |
| Atypia | Mild/Mod. | - | - | 631 | 180 | - | - | - | - | - | - | - | - | - | - | - | - | - | - | 39 | 14 |
|  | Severe | - | - | 88 | 139 | - | - | - | - | - | - | - | - | - | - | - | - | - | NA | 26 | 53 |

**Abbreviation:** TB: tumor budding

**Supplementary Table 5. Summary of a meta-analysis evaluating the relationship of tumor budding with clinicopathological parameters of lung cancer.**

| **Parameters** | **Number of studies** | **Number of patients** | **Pooled OR**  **(95% CI)** | ***P* value** | **Heterogeneity** | | |
| --- | --- | --- | --- | --- | --- | --- | --- |
|  |  |  |  |  | **I^2^ (%)** | ***P* value** | **Model** |
| Age  (<70 vs. ≥70 years old) | 2 | 398 | 0.99 (0.65–1.50) | 0.960 | 0% | 0.760 | Fixed |
| Gender  (male vs. female) | 10 | 3205 | 1.16 (0.98–1.38) | 0.080 | 68% | <0.001 | Fixed |
| Tumor size  (≤30 vs >30 mm) | 3 | 447 | 1.72 (1.14–2.58) | 0.009 | 14% | 0.009 | Fixed |
| Tumor grade (mild moderate vs severe) | 4 | 806 | 0.85 (0.63–1.14) | 0.280 | 4% | 0.370 | Fixed |
| T stage (1+2 vs 3+4) | 4 | 1293 | 3.12 (2.30–4.24) | <0.001 | 78% | 0.003 | Fixed |
| Lymph node metastasis (Negative vs. Positive) | 9 | 2337 | 2.50 (2.04–3.07) | <0.001 | 45% | 0.070 | Fixed |
| Distant metastasis  (Negative vs. Positive) | 3 | 1027 | 1.83 (0.65–5.11) | 0.250 | 0% | 0.630 | Fixed |
| Pathological stage  (I+II vs. III+IV) | 5 | 1337 | 2.68 (1.95–3.69) | <0.001 | 65% | 0.020 | Fixed |
| Pleural Invasion  (Negative vs. Positive) | 6 | 2301 | 3.19 (2.58–3.94) | <0.001 | 65% | 0.010 | Fixed |
| Lymphatic invasion  (Negative vs. Positive) | 6 | 2240 | 4.13 (3.38–5.04) | <0.001 | 36% | 0.170 | Fixed |
| Vascular invasion  (Negative vs. Positive) | 5 | 1755 | 4.83 (3.86–6.05) | <0.001 | 88% | <0.001 | Fixed |
| Nuclear atypia  (mild+moderate vs severe) | 2 | 1069 | 10.62 (7.69–14.68) | <0.001 | 70% | 0.070 | Fixed |
| Smoking  (never vs ever) | 3 | 1436 | 1.39 (1.02–1.89) | 0.040 | 0% | 0.990 | Fixed |

**Abbreviation:** CI: confidence interval, OR: odd ratio

**Supplementary Table 6. Summary of included studies that evaluate publication bias.**

| **Parameters** | **Number of studies** | **Publication bias (*P -* value)** | | |
| --- | --- | --- | --- | --- |
|  |  | **Begg’s test** | **Egger’s test** | **Model** |
| OS | 9 | 0.117 | 0.013 | Fixed |
| Gender (Male vs Female) | 10 | 1.000 | 0.823 | Fixed |
| Tumor size (≤30 vs >30 mm) | 3 | 0.296 | 0.164 | Fixed |
| Tumor grade  (mild moderate vs severe) | 4 | 1.000 | 0.693 | Fixed |
| T stage (1+2 vs 3+4) | 4 | 0.089 | 0.008 | Fixed |
| Lymph node metastasis  (Negative vs. Positive) | 9 | 0.602 | 0.909 | Fixed |
| Distant metastasis  (Negative vs. Positive) | 3 | 1.000 | 0.882 | Fixed |
| Pathological stage  (I+II vs. III+IV) | 5 | 0.462 | 0.180 | Fixed |
| Pleural Invasion  (Negative vs. Positive) | 6 | 0.452 | 0.047 | Fixed |
| Lymphatic invasion  (Negative vs. Positive) | 6 | 0.452 | 0.517 | Fixed |
| Vascular invasion  (Negative vs. Positive) | 5 | 0.462 | 0.145 | Fixed |
| Smoking (never vs. ever) | 3 | 1.000 | 0.387 | Fixed |

**Abbreviation:** OS: Overall survival

**References**

1. Taira T, Ishii G, Nagai K, Yoh K, Takahashi Y, Matsumura Y, et al. Characterization of the immunophenotype of the tumor budding and its prognostic implications in squamous cell carcinoma of the lung. *Lung Cancer*. (2012) 76(3):423-30. doi: 10.1016/j.lungcan.2011.11.010

2. Masuda R, Kijima H, Imamura N, Aruga N, Nakamura Y, Masuda D, et al. Tumor budding is a significant indicator of a poor prognosis in lung squamous cell carcinoma patients. *Mol Med Rep*. (2012) 6(5):937-43. doi: 10.3892/mmr.2012.1048

3. Kadota K, Nitadori J, Woo KM, Sima CS, Finley DJ, Rusch VW, et al. Comprehensive pathological analyses in lung squamous cell carcinoma: single cell invasion, nuclear diameter, and tumor budding are independent prognostic factors for worse outcomes. *J Thorac Oncol*. (2014) 9(8):1126-39. doi: 10.1097/jto.0000000000000253

4. Weichert W, Kossakowski C, Harms A, Schirmacher P, Muley T, Dienemann H, et al. Proposal of a prognostically relevant grading scheme for pulmonary squamous cell carcinoma. *Eur Respir J*. (2016) 47(3):938-46. doi: 10.1183/13993003.00937-2015

5. Zhao Y, Shen H, Qiu C, Zhang T, Hu P, Qu X, et al. Invasion Types Are Associated With Poor Prognosis in Lung Squamous Carcinoma Patients. *Medicine (Baltimore)*. (2015) 94(43):e1634. doi: 10.1097/md.0000000000001634

6. Kadota K, Miyai Y, Katsuki N, Kushida Y, Matsunaga T, Okuda M, et al. A Grading System Combining Tumor Budding and Nuclear Diameter Predicts Prognosis in Resected Lung Squamous Cell Carcinoma. *Am J Surg Pathol*. (2017) 41(6):750-60. doi: 10.1097/pas.0000000000000826

7. Neppl C, Zlobec I, Schmid RA, Berezowska S. Validation of the International Tumor Budding Consensus Conference (ITBCC) 2016 recommendation in squamous cell carcinoma of the lung—a single-center analysis of 354 cases. *Modern Pathology*. (2020) 33(5):802-11. doi: 10.1038/s41379-019-0413-7

8. Yamaguchi Y, Ishii G, Kojima M, Yoh K, Otsuka H, Otaki Y, et al. Histopathologic features of the tumor budding in adenocarcinoma of the lung: tumor budding as an index to predict the potential aggressiveness. *J Thorac Oncol*. (2010) 5(9):1361-8. doi: 10.1097/JTO.0b013e3181eaf2f3

9. Kadota K, Yeh YC, Villena-Vargas J, Cherkassky L, Drill EN, Sima CS, et al. Tumor Budding Correlates With the Protumor Immune Microenvironment and Is an Independent Prognostic Factor for Recurrence of Stage I Lung Adenocarcinoma. *Chest*. (2015) 148(3):711-21. doi: 10.1378/chest.14-3005

10. Enderle-Ammour K, Bader M, Ahrens TD, Franke K, Timme S, Csanadi A, et al. Form follows function: Morphological and immunohistological insights into epithelial–mesenchymal transition characteristics of tumor buds. *Tumor Biology*. (2017) 39(5):1010428317705501.

11. Vasilijević M, Lovrenski A, Panjković M. Tumor budding in tumor tissue among operatively treated patients with lung adenocarcinoma. *Vojnosanitetski Pregled*. (2021) 78(4):409-14. doi: 10.2298/VSP190522091V
